# EpCAM-Mediated Cellular Plasticity Promotes Radiation Resistance and Metastasis in Breast Cancer

**DOI:** 10.3389/fcell.2020.597673

**Published:** 2021-01-06

**Authors:** Arijit Mal, Amirali B. Bukhari, Ram K. Singh, Aastha Kapoor, Amlan Barai, Ishan Deshpande, Tabassum Wadasadawala, Pritha Ray, Shamik Sen, Abhijit De

**Affiliations:** ^1^Molecular Functional Imaging Laboratory, ACTREC, Tata Memorial Centre, Navi Mumbai, India; ^2^Life Science, Homi Bhabha National Institute, Mumbai, India; ^3^Imaging Cell Signaling & Therapeutics Lab, ACTREC, Tata Memorial Centre, Navi Mumbai, India; ^4^Department of Biosciences and Bioengineering, IIT Bombay, Mumbai, India; ^5^Radiation Oncology Unit, ACTREC, Tata Memorial Centre, Navi Mumbai, India

**Keywords:** EpCAM, breast cancer, radiation resistance, cancer stem cell, metastasis

## Abstract

Substantial number of breast cancer (BC) patients undergoing radiation therapy (RT) develop local recurrence over time. During RT therapy, cells can gradually acquire resistance implying adaptive radioresistance. Here we probe the mechanisms underlying this acquired resistance by first establishing radioresistant lines using ZR-75-1 and MCF-7 BC cells through repeated exposure to sub-lethal fractionated dose of 2Gy up to 15 fractions. Radioresistance was found to be associated with increased cancer stem cells (CSCs), and elevated EpCAM expression in the cell population. A retrospective analysis of TCGA dataset indicated positive correlation of high EpCAM expression with poor response to RT. Intriguingly, elevated EpCAM expression in the radioresistant CSCs raise the bigger question of how this biomarker expression contributes during radiation treatment in BC. Thereafter, we establish EpCAM overexpressing ZR-75-1 cells (ZR-75-1^EpCAM^), which conferred radioresistance, increased stemness through enhanced AKT activation and induced a hybrid epithelial/mesenchymal phenotype with enhanced contractility and invasiveness. In line with these observations, orthotopic implantation of ZR-75-1^EpCAM^ cells exhibited faster growth, lesser sensitivity to radiation therapy and increased lung metastasis than baseline ZR-75-1 cells in mice. In summary, this study shows that similar to radioresistant BC cells, EpCAM overexpressing cells show high degree of plasticity and heterogeneity which ultimately induces radioresistant and metastatic behavior of cancer cells, thus aggravating the disease condition.

## Introduction

Breast cancer (BC) continues to be the most common cancer diagnosed among women worldwide. Fractionated ionizing radiation is a standard treatment procedure in BC clinic, which is often used as adjuvant with surgery or chemotherapy. Therapy resistance, i.e., relapsed disease, and metastasis have remained as an unsolved clinical challenge and are major reasons of mortality across all cancers. It has been frequently observed that when subjected to a sub-lethal dose of fractionated γ-radiation, a tumor gradually acquires resistance (Pearce et al., 2001), indicating adaptive radioresistance. Such an adaptive response can be designated as an intrinsic property of a subpopulation of cells, leading to the concept of tumor heterogeneity. The intrinsically radioresistant subpopulation of BC cells are thought to be composed of cancer stem cell (CSC) in the population (Arnold et al., 2020). However, the role of CSC in the acquirement of resistance against radiotherapy (RT) load in BC can not be overlooked. Other than intrinsic radioresistant subpopulations getting selected, it is possible that a subpopulation of cells with altered molecular and phenotypic properties in the tumor mass can overcome the treatment stress, and eventually show resistance to RT. In line to this, it has been reported that radiation can reprogramme non-CSCs to radioresistant CSCs (Lagadec et al., 2012). The question that is also pertinent here is whether any specific stem cell marker/regulator can drive the potential enough to impart radioresistance. Likewise, there are reports of increased incidences of metastasis in resistant BC cases, but no evidence of unique mutation signature identified so far. Thus, the wheel tilts more toward a dynamic cellular adaptive response like radiation induced metastasis of BC cells or selection of a highly metastatic clones, as plausible reasons behind increased cases of metastasis. In this regard, the cellular plasticity concept has evolved against various therapeutic assaults (Elshamy and Duhe, 2013; Arnold et al., 2020; Kong et al., 2020). We have been investigating on aspects of BC radioresistance with an aim to resolve the dichotomy between the purpose and outcome of RT protocol (Desai et al., 2018). As targeting CSC has its own challenges (Kuhlmann et al., 2016; Dashzeveg et al., 2017), identification of a key regulator, which is also a characteristic marker of CSC, contributing to BC radioresistance may potentially provide a new therapeutic option for BC radioresistance in future. In this perspective, we thought of investigating epithelial cell adhesion molecule (EpCAM) as a potential molecule.

EpCAM is a CSC surface marker that was initially discovered as a dominant antigen in colon carcinomas (Herlyn et al., 1979; Visvader and Lindeman, 2008). Recent findings have shown the oncogenic potential of EpCAM and its link to bad prognosis in many cancer types (van der Gun et al., 2010). EpCAM is reported to have a role in radioresistance and chemoresistance in prostate cancer (Ni et al., 2013). It is also reported that EpCAM regulates stemness in nasopharyngeal carcinoma (Wang et al., 2018). EpCAM is reported to be involved in BC metastasis (Osta et al., 2004; Gao et al., 2015; Hiraga et al., 2016). EpCAM is also used as a marker for the detection of circulating tumor cells (CTCs) (Gires et al., 2020). It has been observed that EpCAM high CTCs in metastatic BC are associated with poor overall survival (de Wit et al., 2018). Further, EpCAM high CTCs have shown enhanced metastatic potential than its counterpart (Liu et al., 2019). In nasopharyngeal carcinoma role of EpCAM in metastasis is evaluated *in vivo* (Wang et al., 2018). Thus far, the contribution of EpCAM in BC cellular plasticity, and altered phenotypic regulations such as radioresistance, stemness and further how these phenotypes ultimately impact the metastatic property of cancer cells is not yet established. Therefore, in this study, we focus on relating the contribution of EpCAM in determining altered cellular phenotype both *in vitro* and *in vivo* using experimental radioresistant cell model as well as EpCAM overexpressing condition in BC cells.

## Results

### Radioresistant Breast Cancer Cell Lines Exhibit Altered Focal Adhesion and EMT Profile

**FIGURE 1:**
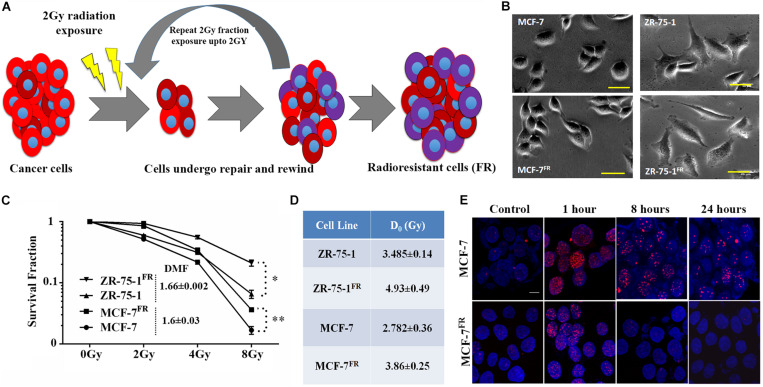
Establishment of radioresistant breast cancer cell model. **(A)** Schematic representation of the radioresistant cell model (FR) established, which shows altered phenotype. **(B)** Photomicrograph of MCF-7^FR^, ZR-75-1^FR^, and their parental counterpart (scale bar-50 μm). **(C)** Long term clonogenic cell survival assay showing the survival fraction of ZR-75-1^FR^, MCF-7^FR^, and their parental counterparts after exposure to single fraction 2Gy, 4Gy, and 8Gy radiation. Dose modifying factor for the respective celllines are mentioned in the inset. **(D)** Table indicates the *D*_0_ values obtained based on cell survival data. **(E)** Immunofluorescence of γH2AX (red) in MCF-7 and MCF-7^FR^ at different time points (1, 8, and 24 h) after 2Gy radiation exposure. DAPI cross-staining is done to mark the nuclei (blue). *P < 0.05, **P < 0.01.

RT protocol in a clinical setting treats the tumor with multiple fractions of γ-radiation, typically 2Gy fractions (Wang et al., 2019). Radioresistant BC lines (FR) were developed *in vitro* by exposing MCF-7 and ZR-75-1 cells to 2Gy γ-radiation for 10 and 15 fractions, respectively (accumulated dose of 20Gy and 30Gy respectively) (Figure 1A). As the cells acquire radioresistance, a morphometric alteration was noticed in MCF-7^FR^ line, which was having a smaller surface area than its parental counterpart (Figure 1B and Supplementary Figure 1A). But in ZR-75-1^FR^ line no such apparent morphological change was observed. The established cell lines, MCF-7^FR^, and ZR-75-1^FR^ were further tested for their survival ability against bulk doses of 2Gy, 4Gy, and 8Gy γ-radiation (Figure 1C and Supplementary Figure 1B). Further, dose modifying factor (DMF) of ∼1.6 in ZR-75-1^FR^ and MCF-7^FR^ cells shows that cells have acquired radioresistance (Figure 1C). MCF-7^FR^ and ZR-75-1^FR^ cells exhibited higher survival fraction and thus represented a higher resistance index (D_0_ value) as 3.86 and 4.93, respectively. In comparison, the baseline MCF-7 and ZR-75-1 showed D_0_ value as 2.782 and 3.485, respectively (Figure 1D). Additionally, it was observed that radiation exposure induced lesser DSB (DNA double strand break), which was evident from lower numbers of γH2AX foci in the nuclei of MCF-7^FR^ and ZR-75-1^FR^ cells as compared to the baselines respectively (Figure 1E and Supplementary Figures 1C–E).

**FIGURE 2:**
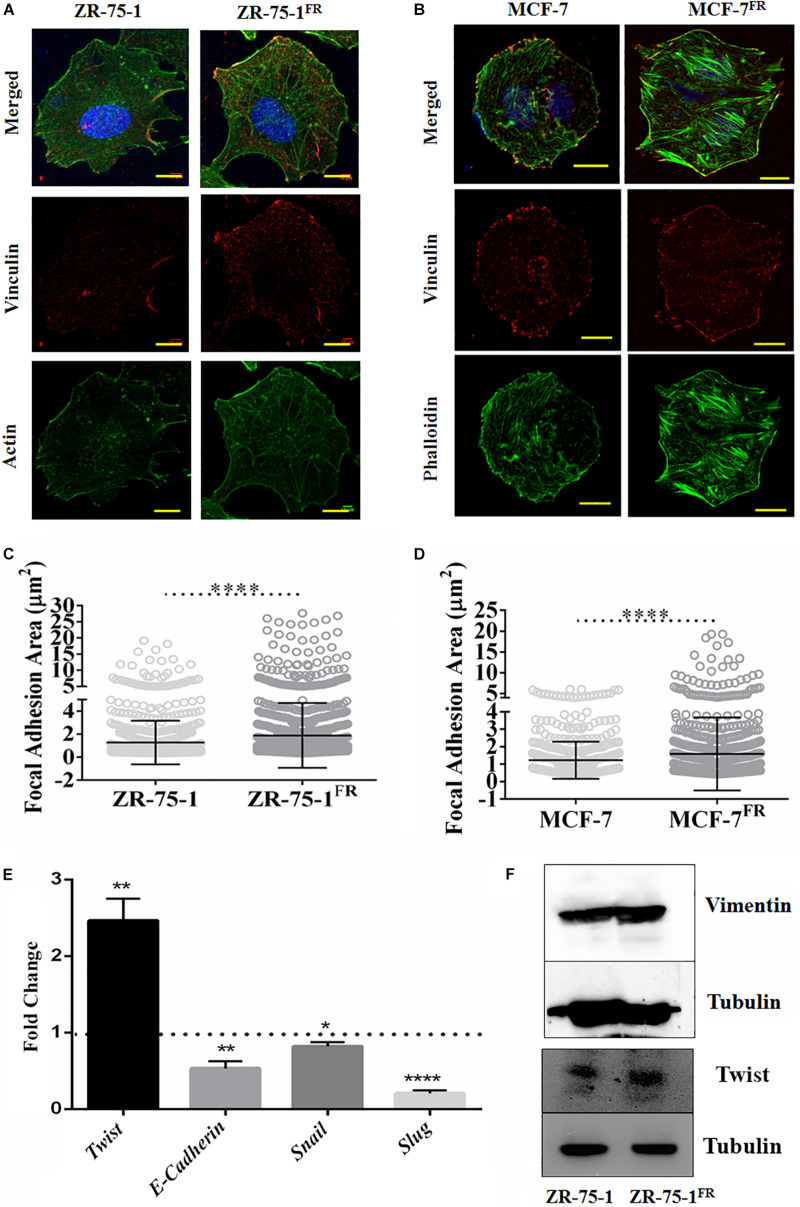
Effect of acquired radioresistance on the focal adhesion and EMT gene expression. **(A,B)** Immunofluorescence photomicrograph of ZR-75-1, MCF-7 cells as well as their radioresistant counterpart after vinculin (red) and F-actin (green) staining. scale bar-10 μm. **(C,D)** Graphs are representing focal adhesion area (μm^2^) measured in ZR-75-1, MCF-7, and their radioresistant counterparts. Focal adhesion area is the co-localization of vinculin and F-actin/phalloidin at the membrane. Each dot in the graph represents the area of individual focal adhesions. **(E)** Graph showing the relative fold change in the mRNA expression of Twist, *E*-cadherin, Snail, and Slug when compared between ZR-75-1^FR^ and ZR-75-1 cells. Dotted line represents the level in ZR-75-1 cells. **(F)** Western blots showing the level of vimentin and Twist in ZR-75-1 and ZR-75-1^FR^ cells, tubulin is used as the loading control. *P < 0.05, **P < 0.01, and ****P < 0.0001.

Ostensive role of focal adhesion proteins in cell migration is well implicated in the literature (Kim and Wirtz, 2013). Co-immunofluorescence staining for vinculin and F-actin showed that MCF-7^FR^, and ZR-75-1^FR^ cells have significantly higher focal adhesion areas than the respective baseline cells (Figures 2A–D). Measured focal adhesion area is the area of co-localization of vinculin and F-actin/phalloidin. Previously, we have reported that radioresistant MCF-7 cells showing mesenchymal phenotype (Desai et al., 2018). Thus, we wanted to investigate the same in ZR-75-1^FR^ cells. We measured the transcript level of important mesenchymal markers such as *Twist*, *Snail*, *Slug*, and epithelial marker *E-cadherin* in ZR-75-1^FR^ cell and represented the relative quantity of transcript by comparing with ZR-75-1 cell (Figure 2E). A two-fold increase in *Twist* transcript was observed, which is a major transcriptional driver of genes expressed in mesenchymal state. We also observed increase in the Twist protein level in ZR-75-1^FR^ cells. However, vimentin expression remain unchanged in between in ZR-75-1^FR^ and ZR-75-1 cells (Figure 2F). These results along with a downward trend in the *E-cadherin* level indicates that the ZR-75-1^FR^ cells started losing epithelial phenotypes while showing an incomplete mesenchymal phenotype. Therefore, ZR-75-1^FR^ cells tilts toward being mesenchymal but have not completely transformed into mesenchymal cells.

### Cancer Stem Cell Features Are Integral to Acquired Radioresistant Phenotype of BC Cells

**FIGURE 3:**
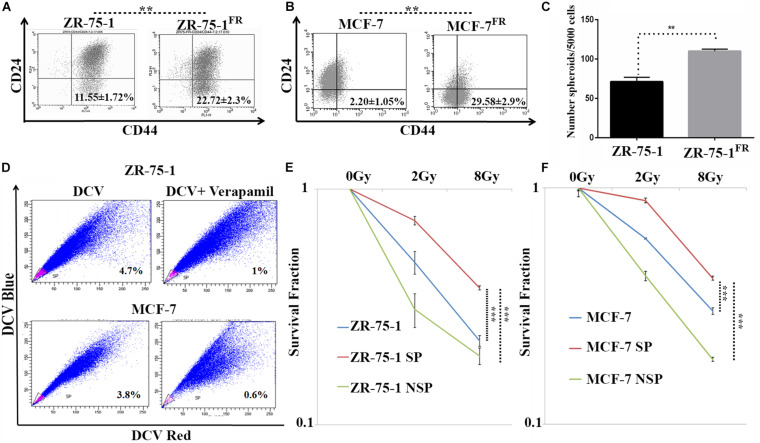
CSCs are closely linked with acquired radioresistance phenotype in BC cells. **(A,B)** Flow cytometry data represents the BCSC biomarker CD24^Low^ and CD44^High^ (lower right quadrant) in ZR-75-1 and MCF-7 and their radioresistant counterpart, respectively. **(C)** Graph represents the number of spheroids formed by ZR-75-1 and ZR-75-1^FR^ cells during mammosphere assay. Statistical significance is calculated by Student’s *t*-test; **P < 0.01. **(D)** Charts representing the percentage of side population (SP) sorted out from ZR-75-1 and MCF-7 cell lines. SP is represented within the gated area in pink color and the non-SP (NSP) cells were in blue color. **(E,F)** Graphs showing survival potentials of SP, NSP and baseline population of ZR-75-1 and MCF-7 cells when exposed to 2Gy and 8Gy radiations. ***P < 0.001.

Published literature has shown that a shift from epithelial phenotype to mesenchymal phenotype is integral to stemness characteristics in acquired radioresistance of BC (Liao and Yang, 2020). The CSC features of established radioresistant cell lines were compared to that of the baseline cells. The radioresistant ZR-75-1^FR^ and MCF-7^FR^ cells showed around 2-fold and 10-fold higher CD44^+^/CD24^–^ population than their baseline counterparts respectively, indicating significant enrichment of CSCs (Figures 3A,B). Additional experimental evidence such as increased number of mammosphere formation (Figure 3C) also complemented the presence of higher numbers of CSCs in radioresistant lines. CSCs can also be identified by a functional assay, called side population (SP) assay. This assay is based on the CSC’s capacity to efflux fluorescent dyes such as DCV or Hoescht at higher rate than non-CSCs. Elevated expression of ATP binding cassette transporter (ABCT) family proteins in CSCs is responsible for the efflux. These SP cells can be isolated by flow cytometry based on decreased fluorescence. Verapamil is used to validate that the decrease of fluorescence in SP cells is due to efflux only. It blocks the efflux pumps, and in turn, there is a decrease in the SP cells as the dye is retained (Wolmarans et al., 2018). We observed that as the BC cells acquired radioresistance, there is an increase in the SP cells (Supplementary Figure 2A). We also observed increased number of ALDH positive cells in the radioresistant population (Supplementary Figure 2B). Taken together these results confirm increased number of CSC when BC cells acquire radioresistance. With increased CSCs in the cell population, radioresistant BC cells showed increased heterogeneity. Further, we planned to verify if the CSCs population display higher survival against γ-radiation. By isolating the SP and non-SP (NSP) population from cell sorter (Figure 3D), we performed a clonogenic survival assay after exposing both radioresistant and baseline ZR-75-1 and MCF-7 cells to a single fraction of 2Gy and 8Gy radiation dose (Figure 3E,F and Supplementary Figures 2F,G). The SP population of both cell lines showed significantly higher survival capacity than the respective baseline or NSP population. Enhanced mammosphere forming capability of the SP cells over NSP cells validated the stemness phenotype (Supplementary Figure 2C). Survival assessment results were further supported by a lesser degree of DNA double-strand breaks (DSB) in SP cells determined by γ-H2AX foci assessment (Supplementary Figures 2D,E). Taken together these results indicate that BC cells when exposed to repeated low dose of RT, may turn heterogeneous with an increase of BCSC in the cell population, a subpopulation of cells that likely help to defy the therapeutic assault.

### EpCAM Overexpressing BC Cells Show Enhanced Ability to Withstand Radiation Stress

**FIGURE 4:**
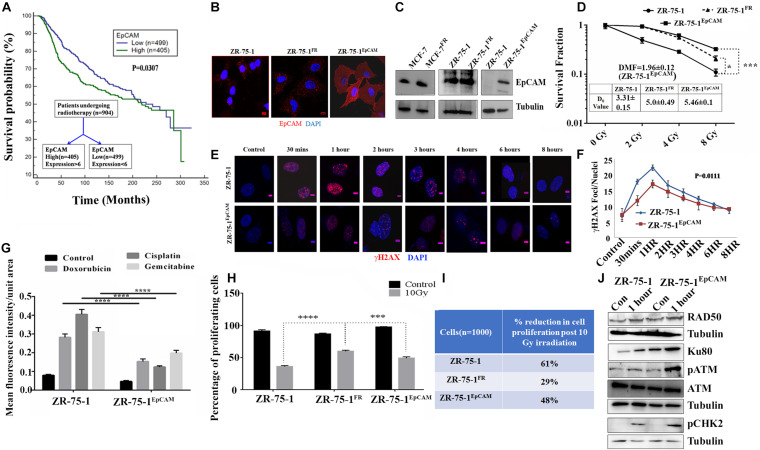
Association of EpCAM with BC radioresistance. **(A)** Kaplan Meier graph showing the overall survival difference between the EpCAM high and EpCAM low groups of 904 patients who underwent radiotherapy in the METABRIC dataset. **(B)** Immunofluorescence photomicrograph showing EpCAM expression (red) in ZR-75-1, ZR-75-1^FR^, and ZR-75-1^EpCAM^ cells, cross-stained with DAPI (blue) (Scale bar-10 μm). **(C)** Semi-quantitative Western blots showing the level of EpCAM. For comparison of EpCAM level between MCF-7 and MCF-7^FR^ as well as ZR-75-1 and ZR-75-1^EpCAM^, 40 μg of protein was loaded. While in case of ZR-75-1 and ZR-75-1^FR^ 150 μg of protein was loaded. **(D)** Comparison of survival fraction between ZR-75-1, ZR-75-1^FR^, and ZR-75-1^EpCAM^ cells against different doses of radiation. Inset table indicates radio-resistivity index (*D*_0_ value) for each. Dose modifying factor(DMF) of ZR-75-1^EpCAM^ cells is shown in inset **(E,F)** Immunofluorescence photomicrograph and graphical representation of γH2AX (red) time kinetic in ZR-75-1 and ZR-75-1^EpCAM^ cells (*n* = 45) after 2Gy radiation exposure. The nucleus is cross-stained with DAPI (blue) (Scale bar-5 μm). Statistical significance between the two groups in **(F)** is determined by Anova. **(G)** Graph showing mean fluorescence intensity of γH2AX foci in ZR-75-1 and ZR-75-1^EpCAM^ cells (*n* = 45) 24 h after treatment with DNA damaging chemotherapeutic drugs, i.e., Doxorubicin (10 μg/ml), Cisplatin (15 μg/ml), and Gemcitabine (60 μg/ml). **(H)** Graph showing the proliferation fraction of ZR-75-1, ZR-75-1^FR^, and ZR-75-1^EpCAM^ cells before and after 10Gy irradiation as determined by EdU proliferation assay. Statistical significance in **(G,H)** is determined by Student’s *t*-test. **(I)** The table represents data derived from the EdU assay as the percentage reduction in cell proliferation in 10Gy irradiated cells over unexposed control. **(J)** Semi-quantitative Western blot showing expression of important molecules of DNA repair pathways involved in untreated and 2Gy radiation treated ZR-75-1 and ZR-75-1^EpCAM^ cells. *P < 0.05, ***P < 0.001, and ****P < 0.0001.

So far, we observed that the established FR cell populations show altered EMT properties and increased BCSC subpopulation. We now ask whether a BCSC specific marker/regulatory protein can contribute to radioresistance in BC. Here, we took interest in the EpCAM protein, which is not only a known marker of BCSC, but is also known to be associated with cellular heterogeneity (Bhatia et al., 2019). Moreover, higher EpCAM transcript and protein was observed in sorted SP cell population than in non-SP population (Supplementary Figures 3A–C). Further, using FACS, we isolated MCF-7 cells with high or low EpCAM expression (Supplementary Figures 4A–D). It was found that EpCAM^Low^ cells are more sensitive to radiation than EpCAM^High^ cells (Supplementary Figure 4E). EpCAM^High^ cells were also able to form higher numbers of mammosphere (Supplementary Figure 4F). Additionally, we have also observed moderate increase of EpCAM level in radioresistant BC cells (Figure 4C). By analyzing a cohort of 904 cancer patients from the METABRIC, TCGA database (Curtis et al., 2012), who underwent RT, it was found that patients with higher than mean value of EpCAM expression show significantly (*P* = 0.0307) inferior overall survival than those with lower EpCAM level (Figure 4A). This data provides an important insight on the clinical association of EpCAM with poor response to RT in BC. Thereafter, to study the functional role of EpCAM in BC radioresistance, EpCAM overexpressing ZR-75-1 (ZR-75-1^EpCAM^) clonal cells were established (Figures 4B,C). In comparison to the parental cells, the ZR-75-1^EpCAM^ cells showed enhanced survival potential similar to ZR-75-1^FR^ radioresistant cells. When ZR-75-1^EpCAM^ cells were exposed to a bolus 8Gy γ-radiation, the survival fraction increased significantly (*P* < 0.001) than the baseline cell (Figure 4D and Supplementary Figure 5A). A high DMF value of 1.96 in EpCAM overexpressing cells also indicates radioresistance due to EpCAM overexpression.

As a measure of acquired radioresistance, it was observed that the FR cell lines display lesser numbers of γH2AX foci formed than their own parental cell line when exposed to 2Gy irradiation. In radioresistant SP cells (with high EpCAM expression), we have observed that γ-radiation induces less DSB (Supplementary Figures 2D,E). Now, by comparing DSB kinetics between ZR-75-1^EpCAM^ and parental cells it was found that the ZR-75-1^EpCAM^ cells have 34, 23, 12, 13, 11, and 10% lower foci than in ZR-75-1 cells at 30 min, 1, 2, 3, 4, and 6 h respectively, thus suggesting that EpCAM overexpressing cells can withstand higher DNA injuries (Figures 4E,F). We have also verified γH2AX foci formation in ZR-75-1^EpCAM^ cells after incubating with other DSB-inducing chemotherapeutic drugs like Doxorubicin (10 μg/ml), Cisplatin (15 μg/ml), and Gemcitabine (60 μg/ml). ZR-75-1^EpCAM^ cells again showed lower numbers of γH2AX foci than parental ZR-75-1 (Figure 4G and Supplementary Figure 5B). Subsequently, it was also observed that radiation exposure have a diminishing effect on DNA synthesis and, in turn, cell proliferation in ZR-75-1^EpCAM^ cells in comparison to its parental counterpart (*P* < 0.001), measured by EdU assay after 10Gy γ-radiation exposure (Figures 4H,I). The cell cycle analysis showed that higher proportions of ZR-75-1 cells are in the G0/G1 phase while the ZR-75-1^FR^ and ZR-75-1^EpCAM^ cells have entered either S phase or G2/M phase after 10Gy irradiation (Supplementary Figure 5G). This indicates that the ZR-75-1^FR^ and ZR-75-1^EpCAM^ cells are undergoing DNA synthesis and is in line with the proliferation data. Further, it was observed that in comparison to ZR-75-1 and ZR-75-1^FR^ cells ZR-75-1^EpCAM^ cells have faster growth rate. When cells were exposed to 2Gy, 4Gy, and 8Gy of radiation, we observed that the ZR-75-1^FR^ and ZR-75-1^EpCAM^ cells have enhanced proliferation rate in comparison to ZR-75-1 cells (Supplementary Figures 5C–F). This imply that radiation have diminished anti-proliferative effect on ZR-75-1^FR^ and ZR-75-1^EpCAM^ cells.

Additionally, we verified the change in expression of key proteins involved in DSB repair in the EpCAM overexpressing cells. Comparing the cell lysates prepared from ZR-75-1 and ZR-75-1^EpCAM^ cells unexposed or exposed with 2Gy radiation, presence of higher Ku80, and Rad50 protein was observed in the radiation exposed ZR-75-1^EpCAM^ cells (Figure 4J). These results indicate a key change in both NHEJ (non-homologous end joining) and HR (homologous recombination) pathway mediated enhanced DSB repair in ZR-75-1^EpCAM^ cells. A higher level of key downstream components such as phosphoATM and its downstream target phosphoCHK2 were noticed in ZR-75-1^EpCAM^ radiation exposed cells. Collectively, the data indicate that the ZR-75-1^EpCAM^ cells are less susceptible to DNA damage against radiation or chemotherapeutic assaults as EpCAM overexpression empowers the DSB repair machinery.

### EpCAM Overexpressing BC Cells Show Enhanced Stemness and Heterogeneity

**FIGURE 5:**
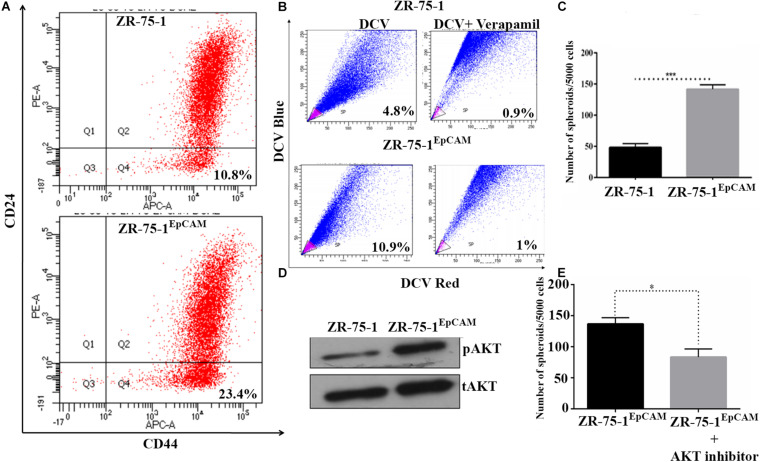
EpCAM overexpression lead to an increase in BCSC subpopulation. **(A)** Flow cytometry analysis for BCSC surface markers CD24^Low^ and CD44^High^ (on lower right quadrant). **(B)** Dot plot showing SP cells (pink) in ZR-75-1 and ZR-75-1^EpCAM^ cell populations. **(C)** Graph showing the quantitative differences in mammosphere formation ability between the cells. **(D)** Western blots showing the level of phosphoAKT (pAKT) and total-AKT (tAKT) in ZR-75-1 and ZR-75-1^EpCAM^ cells. **(E)** Number of spheroids formed in ZR-75-1^EpCAM^ cells treated with or without AKT inhibitor(100 nM). Statistical significance of data in **(C,E)** is determined by Student’s *t*-test. *P < 0.05, ***P < 0.001.

Experimental results obtained so far have shown that radioresistance and BCSC phenotype are intermingled. We have also established that BCSC marker protein EpCAM contributes to radioresistance. These results raise a further question of whether high EpCAM expression can enhance BCSC population in BC cell lines. To address this, we have performed a comparative assessment of CSC between ZR-75-1^EpCAM^ and ZR-75-1 cells. Assessment of CD44^+^/CD24^–^ cell population by flow cytometry showed over two-fold higher CSCs in ZR-75-1^EpCAM^ than ZR-75-1 cells (Figure 5A). This result was further supported by a two-fold higher SP cells in ZR-75-1^EpCAM^ (Figure 5B) and significantly higher numbers of mammospheres (*P* < 0.001) formed in specified culture conditions (Figure 5C). As all the above results indicated a higher CSC population present in EpCAM overexpressing cell line, we further checked for total and phospho-AKT expression, as AKT is one of the key regulators of stemness genes (Rivas et al., 2018). Immunoblots indicate higher activation of AKT in ZR-75-1^EpCAM^ compared to the ZR-75-1 cells (Figure 5D). Thereafter, by incubating ZR-75-1^EpCAM^ cells with an AKT inhibitor, significantly lower (*P* < 0.05) numbers of mammospheres formation were observed in treated condition (Figure 5E). These results affirm that EpCAM overexpression mediated enhanced AKT activation may help in promoting stemness characters in BC cell population.

Cancer stem cells being the tumor initiating cells, cancer cell population with enriched CSCs are expected to form tumor faster by implanting relatively lesser number of cells *in vivo* (Chiang et al., 2017). To verify this, only 100,000 unsorted cells of ZR-75-1^EpCAM^ or ZR-75-1 in the mammary fat-pad were implanted in two sets of mice. By monitoring the tumor growth using non-invasive bioluminescence imaging, it was observed that 3 out of 5 mice in the ZR-75-1^EpCAM^ group and only 1 out of 5 mice in the ZR-75-1 group developed tumor within 30 days (Supplementary Figures 6A–C).

### EpCAM Overexpression Increases Cell Migration Potential in BC

**FIGURE 6:**
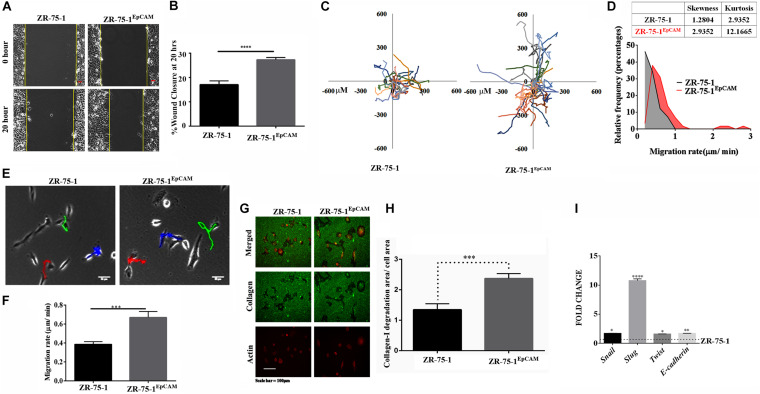
*In vitro* assessment of metastasis potential of EpCAM overexpressing BC cell. **(A,B)** Photomicrograph and quantitative assessment data of wound healing migration assay using ZR-75-1 and ZR-75-1^EpCAM^ are depicting collective migration of cells within 20 h timeframe (scale bar 20 μm). **(C)** Rose plot showing cell migration trajectories. **(D)** Graph comparing the frequency distribution of ZR-75-1 and ZR-75-1^EpCAM^ cells based on their migration rate. Inset table represents skewness and kurtosis determined from the histogram data. **(E)** 2D single cell migration assessment of ZR-75-1 and ZR-75-1^EpCAM^ cells (scale bar 10 μm). **(F)** Graph showing mean single cell migration rate. **(G)** Fluorescence photomicrograph of 3D migration of the two types of cells by invading through the collagen-I ECM matrix. **(H)** Bar graph showing the estimated area of collagen-I matrix invaded by these cells after normalization by the area of the cells. **(I)** Graph showing the relative transcript expression of EMT genes-Twist, Snail, Slug and epithelial gene *E*-cadherin in ZR-75-1 and ZR-75-1^EpCAM^ cells. ***P < 0.001, ****P < 0.0001.

Therapy resistant cancer cells are often reported as a source of metastatic relapse (Zhang et al., 2014). As compared to ZR-75-1, ZR-75-1^EpCAM^ cells showed a significantly higher percent of wound closure (*P* < 0.0001) in scratch wound assay (Figures 6A,B). Further, by tracking single cell migration distance for an average of 50 cells from 3 independent experiments, results reveal that ZR-75-1^EpCAM^ cells traveled longer distances on a 2D matrix than the baseline (Figure 6C). ZR-75-1^EpCAM^ cells showed significantly (*P* < 0.001) higher rate of migration (Figures 6E,F). While the distribution of migration rate (μm/min) was asymmetric for both the cell lines, prominent increase in skewness (a measure of asymmetry) and kurtosis (a measure of peakedness) of ZR-75-1^EpCAM^ cells compared to ZR-75-1 cells is indicative of increase in phenotypic heterogeneity (Figure 6D). Additionally, assessment of collagen-I degradation assessment also showed that ZR-75-1 cells are significantly less invasive than ZR-75-1^EpCAM^ cells (*P* < 0.001) on the extracellular matrix (ECM) (Figure 6G,H). Further, important EMT gene transcripts such as *Snail*, *Slug*, *Twist*, and *E-cadherin* showed significantly higher expression in ZR-75-1^EpCAM^ cells than ZR-75-1, indicating EpCAM overexpression might be transitioning to mesenchymal phenotype (Figure 6I). However, level of *E-cadherin*, an epithelial marker, in ZR-75-1^EpCAM^ cells have not decreased indicating that these cells have still retained the epithelial features. Thus, like ZR-75-1^FR^ cells which are in an intermediate state between epithelial and mesenchymal phenotype, the ZR-75-1^EpCAM^ cells also show nearly similar phenotype. ZR-75-1^EpCAM^ cells tilts toward mesenchymal trait.

**FIGURE 7:**
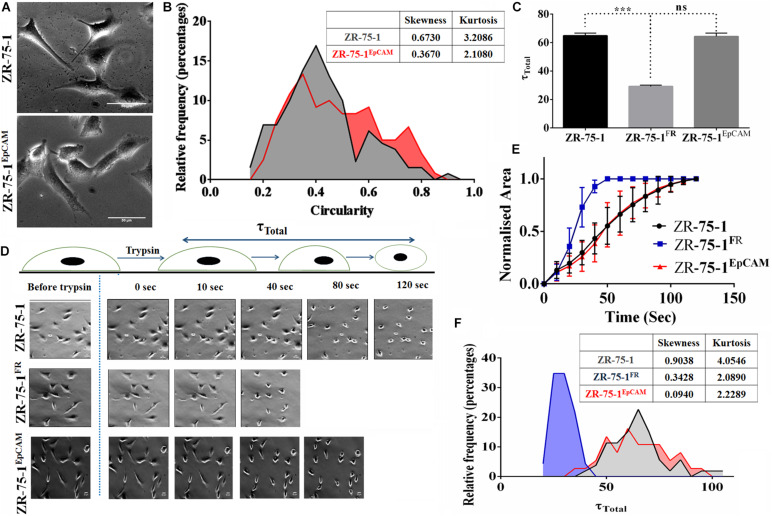
Measurement of cellular morphology and contractility parameters. **(A)** Photomicrograph showing the morphology of ZR-75-1 and ZR-75-1^EpCAM^ cells (scale bar 20 μm). **(B)** Graphs showing the relative frequency distribution of ZR-75-1 and ZR-75-1^EpCAM^ cells based on their circularity. Inset table represents skewness and kurtosis determined from the histogram data. **(C)** Graph representing the total de-adhesion time constant (τ_Total_) of ZR-75-1, ZR-75-1^FR^, and ZR-75-1^EpCAM^ cells. **(D)** Time lapse photomicrograph of de-adhesion kinetics of ZR-75-1, ZR-75-1^FR^, and ZR-75-1^EpCAM^ cells (scale bar 20 μm). **(E)** Graph showing the normalized area of ZR-75-1, ZR-75-1^FR^, and ZR-75-1^EpCAM^ cells during the de-adhesion kinetics as a function of time. **(F)** Graph showing the relative frequency distribution of ZR-75-1, ZR-75-1^FR^, and ZR-75-1^EpCAM^ cells based on their τ_Total_ values. Inset table represents skewness and kurtosis determined from the histogram data. The statistical significance of **(C)** is determined by the Student’s *t*-test. ns, non-signficant, ***P < 0.001.

Cell morphology also provides information on underpinning molecular mechanistics. Differences in the cellular morphology is also reflected in their metatstatic potential. Thus within a cell population tumor cells with varied morphology may have differential metastatic potential (Wu et al., 2020). We have already shown that EpCAM overexpressing cells had higher migration and invasion potential. Moreover, ZR-75-1^EpCAM^ cells have high heterogenous migratory potential. Thus, we looked into the cellular circularity parameter of these cells, a measure of cell roundness. Morphometric analysis of the cells showed a significant difference in the circularity of the cells (Figure 7A). Intriguingly, ZR-75-1 cells showed more homogenous distribution with mean circularity around 0.4, whereas the distribution of ZR-75-1^EpCAM^ represented a wider distribution range of mean circularity with multiple peaks (Figure 7B), which provides further support to their migration potential. Lower kurtosis value of the circularity distribution for ZR-75-1^EpCAM^ cells compared to ZR-75-1 cells further points to the presence of multiple sub-populations in ZR-75-1^EpCAM^ cells.

As actomyosin contractility is an important requirement for enabling cell migration, we have also investigated this aspect. The cellular contractility was measured by trypsin de-adhesion assay. The de-adhesion timescale (τ_Total_) is directly influenced by the actomyosin contractility and is inversely proportional to the cellular contractility (Kapoor et al., 2018). As expected, the ZR-75-1^FR^ cells were found highly contractile and exhibited fastest de-adhesion. Surprisingly, there was no significant difference in de-adhesion timescales of ZR-75-1 or ZR-75-1^EpCAM^ cells (Figures 7C–E). However, points worth noting here is that both skewness and kurtosis values for ZR-75-1^EpCAM^ cells are much lower than ZR-75-1 cells indicating a wider variabilities on τ_Total_ parameter (Figure 7F). EpCAM overexpressing ZR-75-1 cells and ZR-75-1^FR^ cells behaves similarly in many aspects, they are not mirror image of each other, e.g., they show difference in terms of cellular contractility feature. The probable reason might be the acquired radioresistant BC cells consist of multiple clones. These clones might be generated due to radiation induced reprogramming and/or selection of intrinsically radioresistant clone. The established EpCAM overexpressing cell is monoclonal, this is derived from a single cell.

### EpCAM Overexpression Promotes *in vivo* Tumor Growth, Radiation Insensitivity, and Distant Metastasis

**FIGURE 8:**
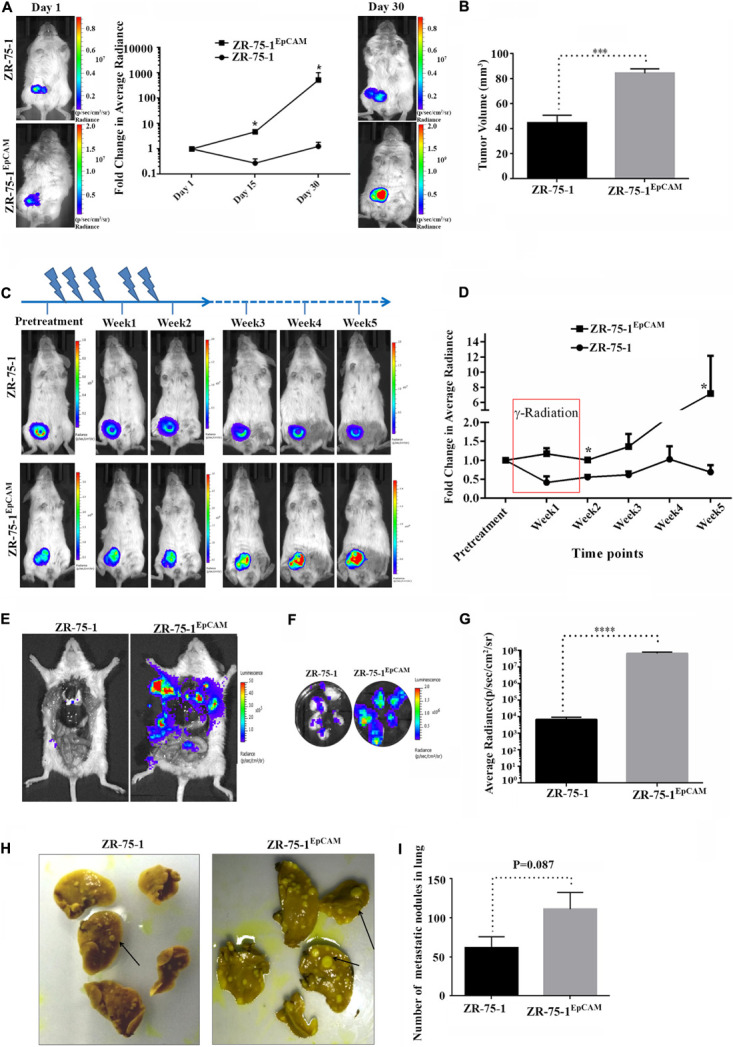
Effect of EpCAM overexpression on *in vivo* tumor growth, RT efficacy and distant metastasis. **(A)** Representative bioluminescence images of mice (*n* = 5) imaged at different time intervals after ZR-75-1 and ZR-75-1^EpCAM^ cell implantation in the mammary fat pad. The graph represents fold change in bioluminescence signal with respect to the signal on day 1. **(B)** Chart showing difference in measured tumor volume on day 30. **(C)** Bioluminescence images showing in vivo growth kinetics of ZR-75-1 and ZR-75-1^EpCAM^ tumors undergoing RT treatment for 2 weeks, as indicated. **(D)** Graph representing fold change in tumor bioluminescence signal with respect to the tumor before treatment. **(E)** Representative images of the ventral view of the mouse from each group dissected and scanned for luciferase signal to show the extent of distant metastasis in major organs. **(F,G)** Bioluminescence images of dissected lung lobes of control and EpCAM overexpressing group and chart showing quantitative photon signal measured from lungs. **(H,I)** Representative photographs of fixed lung tissue with metastatic nodules present at the lung surface (black arrow) and a chart showing numbers of surface nodules counted from these. *P < 0.05, ***P < 0.001, and ****P < 0.0001.

Since EpCAM overexpression leads to enrichment of CSCs and the numbers of CSCs in the cell population positively co-relates with tumor development and growth (Chang, 2016; Papaccio et al., 2017), we investigated the effect of EpCAM expression on *in vivo* tumor development. Firefly luciferase reporter tagged ZR-75-1^EpCAM^ cells exhibited faster tumor growth kinetics than their baseline counterparts over 30 days (Figure 8A). Palpable tumors were observed within 15 days after cell implantation in all 5 mice of ZR-75-1^EpCAM^ group, but only in 3 out of 5 mice of ZR-75-1 group. The ZR-75-1^EpCAM^ cells form a significantly larger volume of the orthotopic tumor (*P* < 0.001) within the timespan of the experiment, indicating EpCAM overexpression promotes *in vivo* tumor growth (Figure 8B).

Further, to validate the *in vitro* findings showing EpCAM overexpression makes BC cells less sensitive to RT treatment, ZR-75-1 and ZR-75-1^EpCAM^ orthotopic tumor bearing mice were subjected to local γ-radiation as outlined in Figure 8C. The tumors were exposed to fractionated 5Gy γ-radiation (25Gy in 5 fractions per week) for 2 weeks, and tumor growth kinetics were followed till 5th weeks using non-invasive bioluminescence imaging (BLI). RT exposure during the first 2 weeks of treatment, ZR-75-1 or ZR-75-1^EpCAM^ tumors showed stable disease as evident from respective BLI scan results. However, the ZR-75-1^EpCAM^ group of mice showed a vigorous tumor growth indicated by ∼8 fold higher (*P* < 0.05) BLI signal than the value observed in ZR-75-1 group (Figures 8C,D). Thus this result validates our *in vitro* finding that that the ZR-75-1^EpCAM^ tumor are less sensitive to radiotherapy than the ZR-75-1 tumors.

Now, therapy resistant cancer cells are likely to be the source of metastatic relapse (Zhang et al., 2014). We have already shown EpCAM overexpressing cells having higher migratory and invasion potential. Moreover the EpCAM overexpression showed increase in E/M hybrid state and enhanced heterogeneity of cell morphology, which in turn is known to show higher metastatic potential as well. Therefore, we further verified the effect of EpCAM overexpression on *in vivo* metastatic potential. The luciferase labeled ZR-75-1^EpCAM^ cells implanted at orthotopic location in a set of mice showed higher BLI signals primarily from the lung area of mice, indicating enhanced secondary tumor growth. Further, confirmation came from the end point, where dissected lung lobes of the ZR-75-1^EpCAM^ group showed significantly (*P* < 0.0001) higher quantity of BLI photon signals than ZR-75-1 group (Figures 8E–G). The dissected lung lobes were further fixed and counted for the number of foci formed with variable size on the surface, which showed nearly two-fold higher numbers of foci in the ZR-75-1^EpCAM^ group than the control group (Figures 8H,I). Further, H and E staining of the metastasized lung tissue showed there is increased number of secondary metastasis for ZR-75-1^EpCAM^ group in comparison to the ZR-75-1 group (Supplementary Figures 7A,B).

## Discussion

Radiotherapy being a practiced treatment modality for BC management, the resistance that develops in patients during the treatment phase is a practical challenge. Due to intra-tumoral heterogeneity in BC, distinct subpopulations of cells responding differently to a given RT load are one of the major obstacles to address this clinical challenge (Hong et al., 2018). Further, the dynamic nature of heterogeneity due to cellular plasticity at different stages of resistance development complicates the matter. The numbers of CSCs within a cell population also varies and part of this dynamicity is known to be contributed by various extrinsic and intrinsic factors (Kong et al., 2020). Here, by isolating CSC subpopulations by cell sorting, we found that they are less susceptible to the radiation (extrinsic factor) induced DNA damage and survive better. After establishing the radioresistant BC cells by repeated exposure to fractionated γ-radiation, our results showed that CSC population directly correlates with the degree of radioresistance, similar to the reported data (Qi et al., 2017). Our study showed that BCSCs subpopulation are intrinsically radioresistant in nature and their proportion goes up within the cell population during the acquirement of radioresistance as well. Further, the established radioresistant cell lines (i.e., ZR-75-1^FR^ and MCF-7^FR^) showed enhanced focal adhesion. Additionally, experimental results based on the ZR-75-1^FR^ cell line showed that BC cells which can survive fractionated γ-radiation of up to 30Gy display higher resistivity index (D_0_), which essentially represents a selected pool with altered focal adhesion, increased heterogeneity and CSC enriched phenotype.

Another novel aspect of this study is the role of an important BCSC marker, EpCAM, identified during BC radioresistance development. Even though the modulation of the cellular heterogeneity in BC cells leading to enhancement of CSC subpopulation occurs, developing therapeutics against CSC has its own challenges (Kuhlmann et al., 2016; Dashzeveg et al., 2017). We considered revealing the role of EpCAM as its contribution in acquired radiation resistance development in BC was unknown. Moreover, its association with cellular plasticity (Bhatia et al., 2019) and the therapeutic targeting strategies for EpCAM is also well developed (Eyvazi et al., 2018). Our results established a positive correlation between high EpCAM level and poor clinical outcome in a retrospective analysis of METABRIC trial patient data who undergone RT. In previous studies, EpCAM was established as an adverse prognostic factor for radiotherapy in head and neck squamous cell carcinoma patients (Murakami et al., 2014, 2019). In the experimental cell line, EpCAM was found to have enhanced expression in BCSCs, which are also radioresistant in nature. Thereafter, by establishing the EpCAM overexpressing ZR-75-1 cell line (ZR-75-1^EpCAM^), we show that its expression can modulate several key molecular features, and thus in turn directs the cells to become radioresistance. Additionally, results of ZR-75-1^EpCAM^ cells also postulated the E/M hybrid status, as it was in ZR-75-1^FR^ as well. Further, the D_0_ value of ZR-75-1^EpCAM^ cell was found higher than the baseline ZR-75-1 cell. Extending the scope of the study, we also showed that the cell fate instated by EpCAM overexpression can significantly impact *in vivo* tumor growth, RT treatment efficacy, and, most importantly, increased metastasis to the distant critical organ. Observed EpCAM mediated faster tumor development and growth may be due to higher number of CSCs present in this cell population as well.

Based on the above experimental results, we see that EpCAM expression is leading to enhanced BCSC subpopulation, causing increased cellular heterogeneity in the cell lines. This made us postulate that EpCAM mediated radioresistance may be due to enhanced stemness. Therefore, we evaluated for activated AKT status and found higher phosphoAKT expression in ZR-75-1^EpCAM^ cell indicating EpCAM regulates stemness through activated AKT as a major path. This finding is in line with a past report wherein nasopharyngeal carcinoma, EpCAM mediated stemness by upregulation AKT/mTOR was demonstrated (Wang et al., 2018). EpCAM is also reported to regulate the AKT/mTOR signaling and, in turn, to be involved in prostate cancer radioresistance (Ni et al., 2013). Findings from the current study affirm similar results for BC. Hence, it seems that EpCAM is a generic marker of radioresistance. However, it will still be worthwhile to study this in the context of various molecular sub-types of BC. With the understanding developed here, future investigation should be aimed at validating these findings in BC patient samples and aim at understanding the contribution of EpCAM during stages of development and maintenance of radioresistance in BC cells.

The ablation of cancer cells by γ-radiation is primarily due to the induction of DSBs (Vignard et al., 2013; Santivasi and Xia, 2014; Kumari et al., 2019). In accordance with the reported literature, radioresistant BC cells, BCSCs, and even the ZR-75-1^EpCAM^ cells are found less susceptible to radiation induced DSBs. EpCAM overexpressing cells, when exposed to radiation, displayed elevated expression of key initiator molecules of the two main DSB repair pathways, i.e., Ku80 for NHEJ and Rad50 for HR pathways. We also found that when compared to the irradiated baseline cells, irradiated EpCAM overexpressing cells markedly activates both ATM and its downstream target CHK2. As per the reported evidences, the phospho-CHK2 further carryout the required signaling machinery and helps the cells to cope with the DNA damage (Jin and Oh, 2019). We also observe that ZR-75-1^EpCAM^ cells, by virtue of its enhanced and active DSB repair machinery, are less affected by the DNA damaging chemotherapeutic agents, such as doxorubicin, cisplatin, or gemcitabine.

Now coming to the point of hybrid E/M status or heterogeneity as a linked feature to enhanced metastatic potential of radioresistant BC cells, our analysis of various EMT markers showed that the ZR-75-1^FR^ cell population has neither completely lost their epithelial phenotype nor they showed enhanced mesenchymal phenotype. As a epithelial cell transitioned to mesenchymal phenotype, decreased expression of epithelial markers with increase in mesenchymal markers was observed. But, most of times cells are not in binary state, either being completely mesenchymal or epithelial (Kroger et al., 2019; Yang et al., 2020). Literature reports suggests occurrence of two way transition, i.e., E to M transition (EMT) and M to E transition (MET) (Liu et al., 2016; Bhatia et al., 2020). Thus, cancer cell population attains a phenotype which falls within the spectrum of epithelial to mesenchymal phenotype. Such is a hybrid state, which is essentially linked to cellular plasticity (Jolly et al., 2015) as well as enhanced survival and collective migration.

Previously, we reported that radioresistant BC cells has higher migratory potential (Desai et al., 2018). The integral part of the cancer cell metastasis process involves invasion and cell migration. Hence, we have thoroughly evaluated the EpCAM overexpressing ZR-75-1 cells for their migratory potentials and found that these cells gain the power of both collective as well as single-cell migration on 2D matrix. EpCAM overexpressing cells are found more heterogeneous than the parental cells with respect to their 2D migration rate. Heterogeneity was also apparent for other cellular phenotypes like cell morphology and cellular contractility. Cancer cells can migrate collectively or as a single cell (Friedl and Wolf, 2010), and migrating cells have to invade through the ECM (extracellular matrix) as well. Here we found that high EpCAM expression positively influences increased invasion in the collagen-I matrix. We also observed enhanced migration and invasion potential of ZR-75-1^EpCAM^ cell subsequently leads to enhanced distant metastasis to the lung from the orthotopic primary site in the immunocompromised mouse model. While it is reported that EpCAM expression correlates to distant metastasis in different patient cohorts, including BC (Osta et al., 2004; Zeng et al., 2019), our study using non-invasive imaging method showed for the first time that EpCAM imparts in metastasis in preclinical settings.

In conclusion, this study reveals the potential of EpCAM during the development of BC radioresistance. BC cells having high expression of EpCAM gain the ability to refract the RT efficacy by promoting cellular heterogeneity, enhanced plasticity within the cancer cell population, and thus pushing the cell fate more toward the CSC phenotype. High EpCAM status can influence the cells to attain a hybrid E/M status and enhance distant metastasis *in vivo*. Considering all published evidence across various cancers so far, EpCAM appears to be a generic marker of radioresistance. Therefore, EpCAM mediated resistance development against RT procedure may be taken into consideration for future evaluation of local recurrence in BC patients.

## Materials and Methods

### Materials

The drugs used were Doxorubicin (Sigma Aldrich, D1515), Cisplatin (Sigma Aldrich PHR 1624) and Gemcitabine (Sigma Aldrich, G6423). D-luciferin imaging substrate from (Biosynth, L-8220). The inhibitor of AKT (CST9901) is obtained from Cell Signaling Technology. EdU click-IT cell proliferation kit from (Thermo Fisher Scientific, C10340). EGF-Epidermal growth factor (Sigma Aldrich E9644), FGF-Fibroblast growth factor (Sigma Aldrich F0291), Insulin (Sigma Aldrich 91077C) and LIF-Leukemia inhibitory factor (Thermo fisher PHC9481).

### Cell Culture

ZR-75-1 cell line (ER^+^, PR^+^, and Her2^Low^) and MCF-7 cell line (ER^+^, PR^+^, and Her2^Low^) was cultured in RPMI 1640 media with recommended supplements and standard culture conditions. EpCAM ORF cloned in pCDNA3.1 mammalian vector is used for generation EpCAM overexpressing ZR-75-1 cells. Briefly, pCDNA3.1-EpCAM transfected ZR-75-1 cells were diluted and plated to obtain monoclonal cell colony arising from single cell. Puromycin (Sigmal Aldrich P8833) was used as a selection pressure. Monoclonal colonies arising from single cells were evaluated for EpCAM expression for multiple passages. The clone showing stable expression of EpCAM for multiple passage was selected for further study. ZR-75-1 and ZR-75-1^EpCAM^ cell populations were further labeled using firefly luciferase by lentiviral transduction using third generation lenitiviral vectors –fluc-tdTomato cloned lentiviral vector, P-delta packaging plasmid, VSVG envelope protein plasmid.

Radioresistant ZR-75-1 and MCF-7 cell lines were generated by fractioned irradiation of 2 Gy every 2 days. After every five fraction of irradiation cells surviving cells were allowed to repopulate and then another round is started.

### TCGA Analysis

From METABRIC dataset, we identified 904 patients undergoing radiotherapy. We then segregated them into two groups based on EpCAM level-EpCAM high, where EpCAM level is higher than the mean expression and EpCAM low, where EpCAM level is lower than the mean expression. Their overall survival was plotted as Kaplan-Meier curve.

### Animal Imaging

All the experiments were approved by the Institutional animal ethics committee of ACTREC, TMC, India, and were performed in accordance with accepted guidelines. 5 × 10^6^ firefly luciferase reporter labeled cells were implanted in mammary fat-pad of 6–8 weeks old SCID mice. Non-invasive bioluminescence imaging (BLI) scan was performed using IVIS Spectrum (Perkin Elmer) after injecting 100 μl D-luciferin substrate (30 mg/ml). Mice were maintained under 2% isoflurane gas anesthesia during the scan. Data analysis was performed using Living Image software v4.4.

### *In vitro* Irradiation

Breast cancer cell lines grown on cell culture plate at 70% confluency were irradiated by SSD (surface to source distance) technique for specified doses using ^60^Co-γ Linear Accelerator, indigenous Bhabhatron machine. D_0_ is the dose at which the survival is 37%. It was determined from the clonogenic assay based on the formula S = e^–αD–βD2^, where S is the survival percentage, D is the single dose to which cells are exposed and α and β are parameters describing the cell’s radiosensitivity. Dose modifying factor(DMF) is the ratio of dose of radioresistance cells to that of its parental counterpart at which survival is 50%.

### *In vivo* Irradiation in Mice

Mice bearing orthotopic tumors were anesthetized and only the tumor was exposed to radiation by shielding the rest of the body using lead blocks. Exposure to accumulated dose of 25Gy was given in five fractions every 3 days over 2 weeks period by SSD technique using ^60^Co-γ Linear Accelerator. The radiation dose schedule chosen for this study was based on the calculation of biological effective dose and its 2 Gy (the conventional daily dose of radiation) equivalent. Mice were irradiated twice a week to allow recovery and repair of damage in adjacent normal tissues. All the mice tolerated the radiation schedule reasonably well without sign of systemic toxicity.

### *In vivo* Metastasis Study

Mice with either ZR-75-1 or ZR-75-1^EpCAM^ cells overexpressing luciferase reporter were implanted at mammary fat pad and monitored by performing weekly BLI scan until signal appeared outside of the primary tumor area. Mice were sacrificed after 90 days using cervical dislocation and major organs were harvested and imaged *ex vivo*. Boudin solution was used for fixing the lungs and the number of metastatic foci on organ surface was counted under a binocular microscope. Hematoxylin and eosin stain of lung was also carried out. The images were captured at 1X, 5X, and 10X magnification using Zeiss upright microscopy.

### Immunoblotting

Cell lysates are prepared using Ripa Buffer and run in SDS-PAGE gel as per standard protocol. After the proteins are transferred in nitrocellulose membrane, blocking was done using 5% BSA. Primary and Secondary antibodies are mixed in 5% BSA. We used primary antibodies of EpCAM (Santa Cruz, sc-25308), phosphoAKT (Sigma Aldrich, SAB5600064), Total AKT (Sigma Aldrich, SAB5600066), Rad50 (Abcam, ab8913). Ku80 (Cell Signaling Technology, CST2180), phosphoATM (Cell Signaling Technology, CST D6H9), Total ATM (Cell Signaling Technology, CST2873), CHK2 (Cell Signaling Technology, CSTC13C1), Vimentin (Sigma Aldrich V6630), Twist (Santa Cruz, sc-15393) and Tubulin (Abcam, ab7291). Primary antibody of EpCAM has dilution of 1:200, while rests of the antibodies have dilution of 1:1000. We used anti-mouse HRP secondary antibody (ab6728) and anti-rabbit secondary antibody (Thermo Scientific 35512) at 1:10000 dilution.

### Immunofluorescence Imaging

Cells were seeded in coverslips (10 × 25 mm). The cells washed thrice with PBS before fixing with 4% paraformaldehyde for 10 min at 37°C. Then cells were again washed thrice in PBS. For nuclear permeabilization in cells, 4% paraformaldehyde containing 0.2% triton X (incubated at room temperature for 10 min) was used. The coverslips are incubated with primary antibody at 4°C, overnight. While the coverslips is incubated with a secondary antibody, which is tagged with anti-rabbit Dylight 633 (Invitrogen 35562) (dilution 1:100) for 1 h at room temperature. The primary antibody used is γH2AX (Cell Signaling Technology, CT20E3, dilution 1:300), EpCAM(Abcam ab8601, dilution 1:50) Vinculin (Abcam ab18058, dilution 1:400) and Phalloidin (Thermo scientific 22287, dilution 1:500). The cell nuclei were stained with DAPI (Sigma Aldrich D9142, 1 mg/ml stock, working dilution 1:200). After washing thrice with PBS, the coverslips were mounted on glass slides using VECTASHEILD (Vector CB 1000). Then the images of around 45–50 cells were captured using Zeiss LSM780 confocal microscope. Images were analyzed in Zeiss LSM780 software.

Focal adhesion area is measured as the area of co-localization of vinculin and F-actin/phalloidin. The area of co-localization was measured using ImageJ.

### Side Population Assay

One million cells are suspended in 1 ml cell culture medium. The negative control group is one which is treated with 50 μM verapamil (Sigma Aldrich V4629) and incubated in 37°C for 30 min. Then 1 μl DCV (Thermo Fisher Scientific V35003) was added in all the groups and incubated at 37°C for 90 min. Then the cells were washed thrice in PBS and re-suspended in PBS for acquisition in flow cytometry.

### BCSC Surface Marker Assessment

1 × 10^6^ cells are harvested and washed thrice with PBS. Then cells are suspended in 50 μl PBS and incubated with CD24 (Sigma Aldrich SAB4700624), and CD44 (Sigma Aldrich SAB4700185) fluorescence tagged primary antibody for 1 h at 37°C at the company recommended at dilution. Then cells are again washed with PBS, and CD24−/CD44+ cells were analyzed by flow cytometry.

### Mammosphere Formation Assay

Five thousands cells are seeded in ultra-low attachment 6 well plate (Corning #3471). The cells are grown in incomplete cell culture medium –RPMI 1640 complemented with FGF (20 ng/ml), EGF (10 ng/ml), Insulin (20 ng/ml), LIF (10 ng/ml) and 0.1% pen-strep and passaged after 8 days till three passages. The spheroids having a size of more than 50 μm are counted under a microscope.

### Wound Healing Assay

Cells are grown to 90% confluency in monolayer. Then a small wound is made. The image of the wound was captured every 1 h in inverted microscopy for 20 h. Then we measured the % wound closure = woundareain 0hour-woundareain 20hourwoundareain 0hour*100

### 2D Cell Migration Assay

20,000–30,000 cells were seeded in six well plates. Then the cells were imaged at 20X every 20 min for 20 h. Then the distance traveled by the cell were collected by measuring the cell trajectories using the manual track plugin in ImageJ. Further determining the speed from the distance traveled.

### Collagen Invasion Assay

Cells are plated on fluorescently labeled rat tail collagen I (concentration of 10 μg/cm^2,^ Sigma, Cat # C3867) coated glass coverslips. Glass coverslips were incubated overnight with collagen I at 4°C to allow passive adsorption. The coverslips were further blocked with 1% Pluronic F127 (Sigma, P2443) for 10 min to prevent non-specific binding. Collagen is stained with mouse polyclonal primary antibodies (Merck, custom made) at 1:500 overnight and counterstained with anti-mouse secondary antibody at 1:1,000 for 2 h. Collagen degradation was assessed after culturing cells for 6 h to allow for visible degradation. Quantification of the degraded area was performed using ImageJ.

### Trypsin Deadhesion Assay

Trypsin de-adhesion assay was carried out as described earlier (Sen and Kumar, 2009). Briefly, the cells were incubated with warm 1X trypsin. Then the image of cells was captured every 1-s using time-lapse microscopy. The area of the cells was measured after the addition of trypsin till the cells become round. Then the area was normalized using the formula

$$An=\frac{Ai-At}{Ai-Af}$$

Where, *An* is normalized area, *Ai* is initial area of the cells just before addition of the trypsin, *Af* and *At* is the final area and area at time *t* of a cell. The normalized area of the cells was plotted as a function of time where the curve was fitted to

$$An=1-\frac{1}{1+e^{-\left(1-\tau 1\right)/\tau 2}}$$

Based on the formula deadhesion time, constant τ was determined.

### Spread Area Analysis

Cells were seeded on 0.1 μg/cm^2^ collagen-coated coverslip and imaged after 24 h incubation using an inverted microscope (Olympus IX71). Then using ImageJ, the area of the cells was measured by manually tracing the perimeter of the cells. Based on the area of the cells the circularity (*C*) of the cells were determined using the formula *C* = 4Π*A*/*p*^2^, where *A* is the area of the cells, and *p* is the perimeter of the cells.

### Clonogenic Survival Assay

Five hundred cells are seeded in six well plates. Then cells are irradiated at different doses. The cells are allowed to forms colonies for 10–14 days. Then the colonies are fixed with 90% methanol at room temperature for 10 min and subsequently stained with crystal violet (Sigma Aldrich C0775). Then the colonies are counted using a stereomicroscope. The surviving fractions are derived following the formula-

PE of the treated sample/PE of the control*100

Where, PE = Number of colonies obtained / Number of cells seeded. *100

### Real Time PCR

RNA was extracted from ZR-75-1 and ZR-75-1^EpCAM^ cells using RNeasy kit (Qiagen-74104). cDNA was synthesized using first-strand cDNA Kit (Thermo Scientific-18080085). Real time PCR was carried out using SYBR Green (Agilent-600828). Primers for EpCAM-Forward primer 5′ GCAGCTCAGGAAGAATGTG 3′ and Reverse primer 5′ CAGCCAGCTTTGAGCAATGAC 3′. That for GAPDH– Forward primer 5′ GAAGGTCGGAGTCAACGGATT 3′ and reverse primer 5′ CCAGACTTAAAAGACCT 3′, *E*-cadherin-Forward primer CTTTGACGCCGAGAGCTACA and reverse primer TTTGAATCGGGTGTCGAGGG, Snail forward primer CCAGTGCCTCGACCACTATG and reverse primer CTGCTGGAAGGTAAACTCTGGA, Slug Forward primer TTCGGACCCACACATTACCT and reverse primer TTCTCCCCCGTGTGAGTTCTA and for Twist forward primer TCTACCAGGTCCTCCAGAGC and reverse primer CTCCATCCTCCAGACCGAGA.

### EdU Cell Proliferation Assay

Cells were incubated for 16 h with EdU after exposure to 10Gy. Then the cells were processed as mentioned in the EdU Assay Kit (Abcam, ab219801).

### Cell Cycle Analysis

Cells seeded at 60–70% confluency were harvested and fixed with 70% ethanol. Ethanol is added dropwise while slowly vortexing the cells. The cells were fixed overnight at −20°C. Then washed with PBS and resuspended in PBS solution containing 100 μg RNAase A and 25 μg propidium iodide for 30 min. Then the DNA content of the cells were analyzed in flowcytometry.

### Growth Curve

Two thousands cells were seeded on day 0. Then every 24 h the cells were harvested and counted to check the growth.

### ALDH Assay

The assay was carried out using AldeRed ALDH Detection Assay (Sigma-Aldrich SCR150). The cells were processed as mentioned in the assay kit.

### Statistical Analysis

Histograms showing frequency distributions was calculated using Graphpad Prism. The skewness and kurtosis of frequency distributions was calculated using third moment and fourth moment tests, respectively. Unpaired Students *t*-test and Anova was used to calculate the statistical significance. *P*-value ≤ 0.05 was considered significant and indicated as ^∗^ ≤ 0.05, ^∗∗^ ≤ 0.01, ^∗∗∗^ ≤ 0.001, and ^****^ ≤ 0.0001. Error bars indicate standard error mean of triplicate biological samples.

## Data Availability Statement

The original contributions presented in the study are included in the article/Supplementary Material, further inquiries can be directed to the corresponding author/s.

## Ethics Statement

The animal study was reviewed and approved by the Institutional Animal Ethics Committee (IAEC), ACTREC, TMC.

## Author Contributions

AD and PR conceived the idea and designed the experiments. AM, AB, RS, AK, AB, and ID performed the experiments. AM, AB, TW, PR, SS, and AD analyzed the data and drafted and edited the manuscript. All the authors contributed to the article and approved the submitted version.

## Conflict of Interest

The authors declare that the research was conducted in the absence of any commercial or financial relationships that could be construed as a potential conflict of interest.
