# Supplementary material for: EpCAM-Mediated Cellular Plasticity Promotes Radiation Resistance and Metastasis in Breast Cancer
Source: Front Cell Dev Biol. 2021 Jan 6;8:597673. doi: 10.3389/fcell.2020.597673 (PMC7815650; doi:10.3389/fcell.2020.597673)
Supplement: Supplementary file 1 [file Data_Sheet_1.pdf]

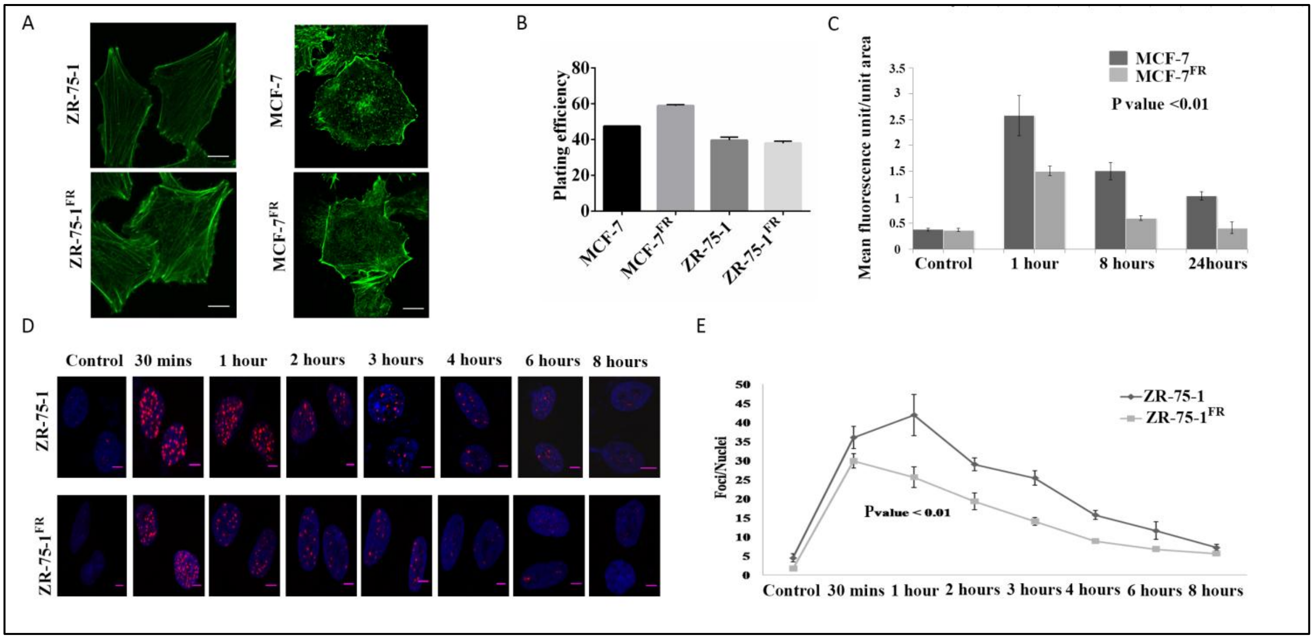

***Supplementary Figure-1- A**- Immunofluoresence photomicrograph of F-Actin staining (green) of ZR-75-1, MCF-7 cells and their radioresistant counterparts. Scale bar- 10µm. **B**- Bar graph showing the platting efficiency. **C**- Bar graph showing the quantification of γH2AX level in MCF-7 and MCF-7^{FR} cells. **D**- Immunofluoresence photomicrograph of γH2AX foci in ZR-75-1 cells and ZR-75-1^{FR} cells at different time points post 2Gy irradiation. Red- γH2AX foci and Blue-DAPI (Scale bar-5µm). **E**- Line graph represents the quantification of γH2AX level from panel D. Statistical significance between the two groups was determine by Anova.*

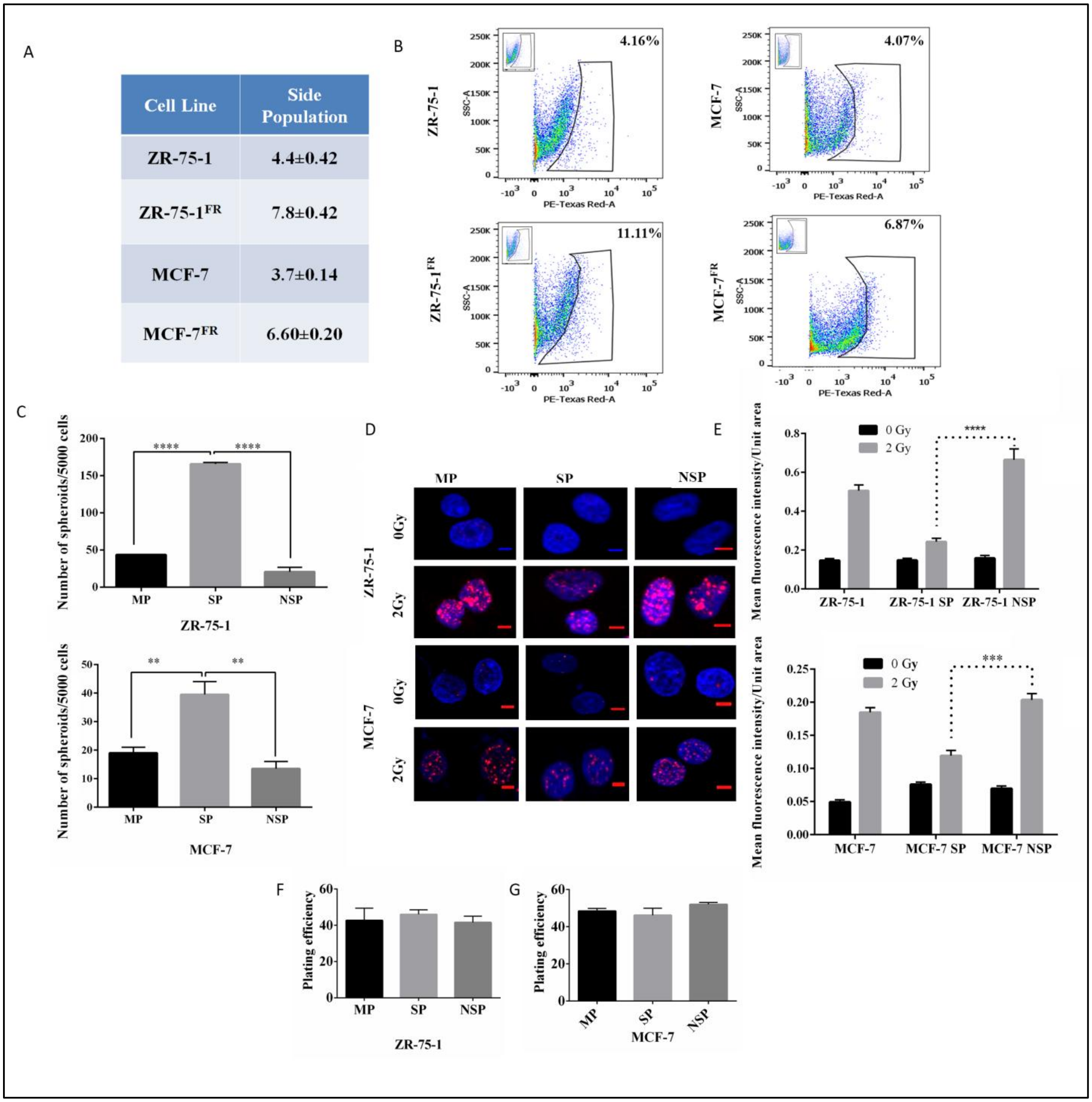

Supplementary Figure-2-A- *Table showing the level of CSC sub-population identified by side population assay.* ***B***- *Dot plot showing the level of ALDH positive cells in ZR-75-1, MCF-7 cells and their radioresistant counterpart. Inset dot plot shows the level of ALDH positive cells in presence of DAEB.* ***C***- *Graph showing the number of spheroids formed by SP, MP and NSP of ZR-75-1 and MCF-7 cells.* ***D***- *Immunofluoresence photomicrograph of γH2AX foci in MP, SP and NSP of ZR-75-1 and MCF-7, 1 hour post 2Gy irradiation, where red indicates γH2AX foci, and blue indicates DAPI (Scale bar-10µm).* ***E***- *Bar graph represents the quantification of γH2AX*

level from panel D. ***F, G****- Bar graph showing the platting efficiency of ZR-75-1 and MCF-7 cells. Statistical significance of data in C and E is determined by Student's t-test. **- P<0.01, ***- P<0.001,****- P<0.0001*

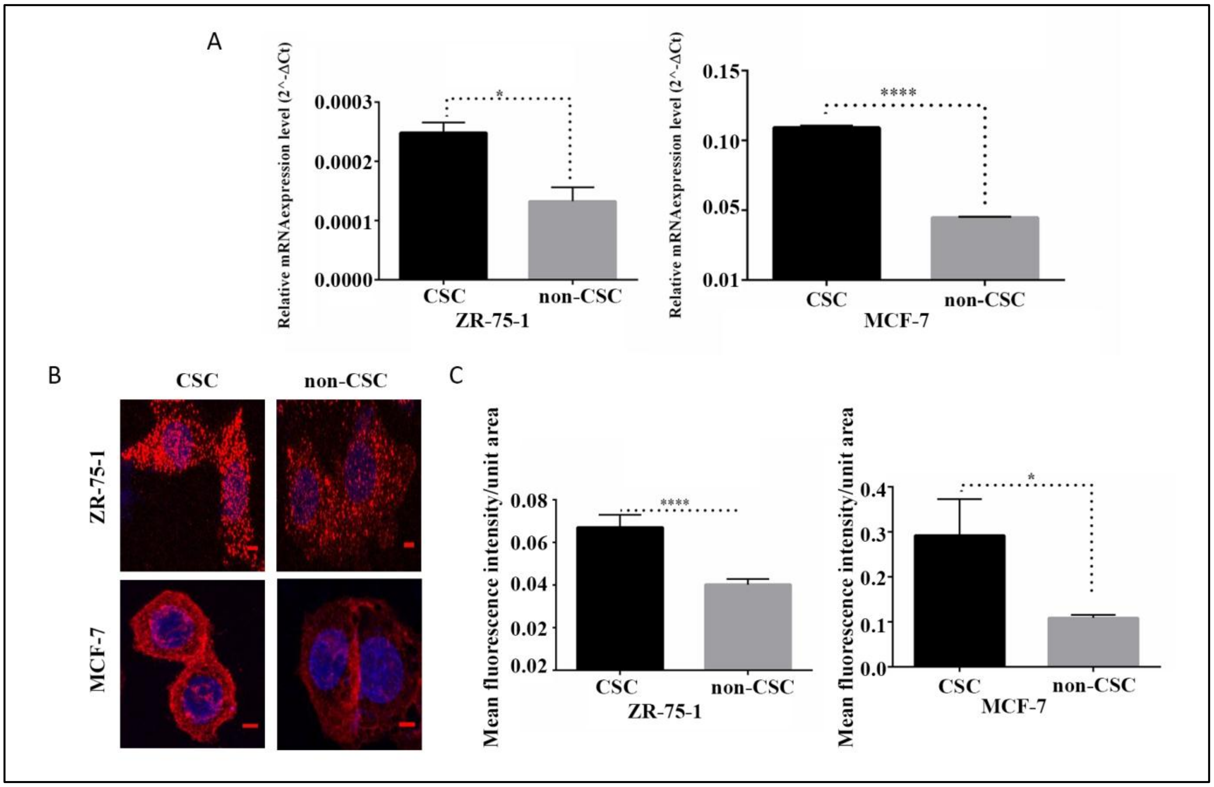

Figure 3*- **A**- Graph representing the relative level of EpCAM mRNA to that of housekeeping gene GAPDH in CSC (SP) and non-CSC (NSP) of ZR-75-1 and MCF-7.* ***B, C****- Immunofluoresence photomicrograph and graphical representation of the level of EpCAM(red) in CSC and non-CSC of ZR-75-1 and MCF-7 where nucleus is represented in blue. Scale bar -5μm. Statistical significance of data in A and C is determined by Student's t-test. *- P<0.05, ****- P<0.0001*

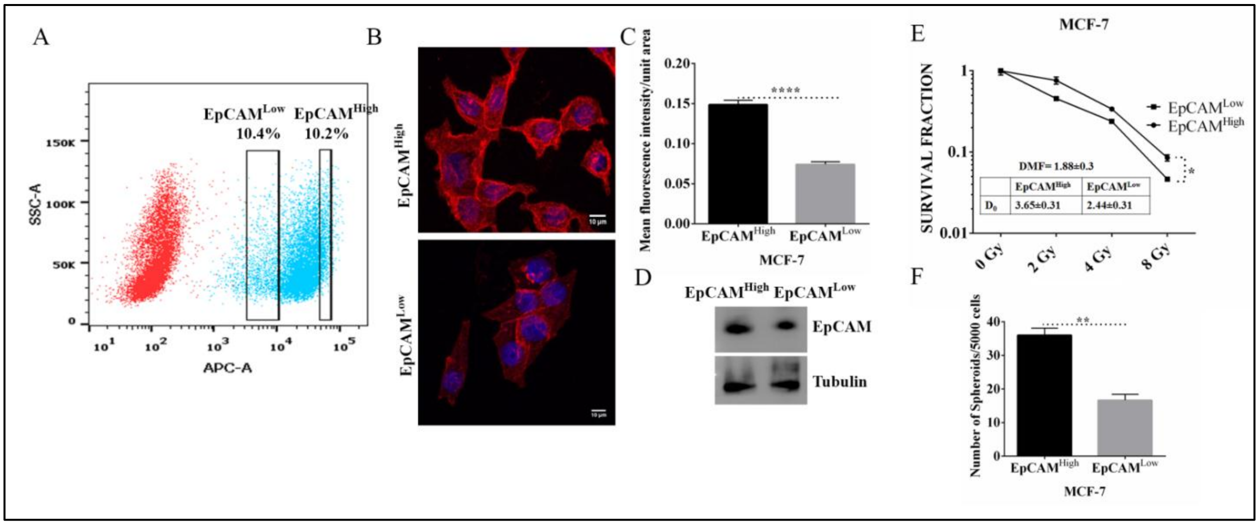

Supplementary figure 4-A*-Dot plot showing the distribution of MCF-7 cells based on the EpCAM detected using flowcytometry. The dot plots in red show the secondary control, while one in blue show the positive control. Cells isolated as EpCAM^{Low} and EpCAM^{High} is shown in black box.* ***B,C****- Immunofluoresence photomicrograph and graphical representation of the level of EpCAM(red) in EpCAM^{Low} and EpCAM^{High} population of MCF-7 cells where nucleus is represented in blue. Scale bar -10µm.* ***D*** *-Semi-quantitative western blot showing expression level of EpCAM in EpCAM^{Low} and EpCAM^{High} population of MCF-7 cells.* ***E****- Comparison of survival fraction between EpCAM^{Low} and EpCAM^{High} population of MCF-7 cells against different doses of radiation. Inset table indicates radio-resistivity index (D_0 value) for each. The dose modifying factor(DMF) is also mentioned in inset. Statistical significance of data in C, E and F is determined by Student's t-test.*- P<0.05, **- P<0.01, ****- P<0.0001*

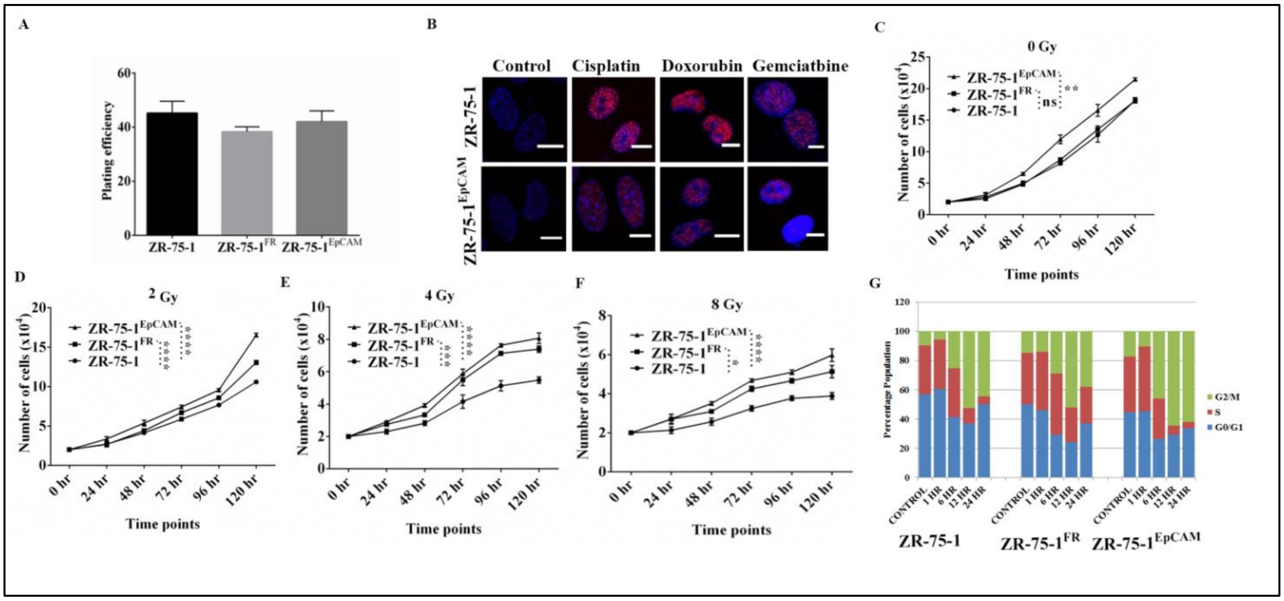

Supplementary Figure*-5-*A- *Bar graph showing the platting efficiency of ZR-75-1, ZR-75-1^{FR} and ZR-75-1^{EpCAM} cells.* ***B***- *-Immunofluoresence photomicrographs of γH2AX foci in ZR-75-1 and ZR-75-1^{EpCAM} cells after treatment with doxorubicin, cisplatin and gemcitabine for 24hours. Scale bar is 10µm.* ***C -F*** *Line graph showing the growth curve without irradiation and on 2Gy, 4Gy and 8Gy irradiation, respectively.* ***G***- *Cell cycle analysis of ZR-75-1, ZR-75-1^{FR} and ZR-75-1^{EpCAM} cells at different time points, post 10Gy irradiation. . Statistical significance of data in C-F is determined by Anova. *- P<0.05, **- P<0.01, ***- P<0.001, ****- P<0.0001*

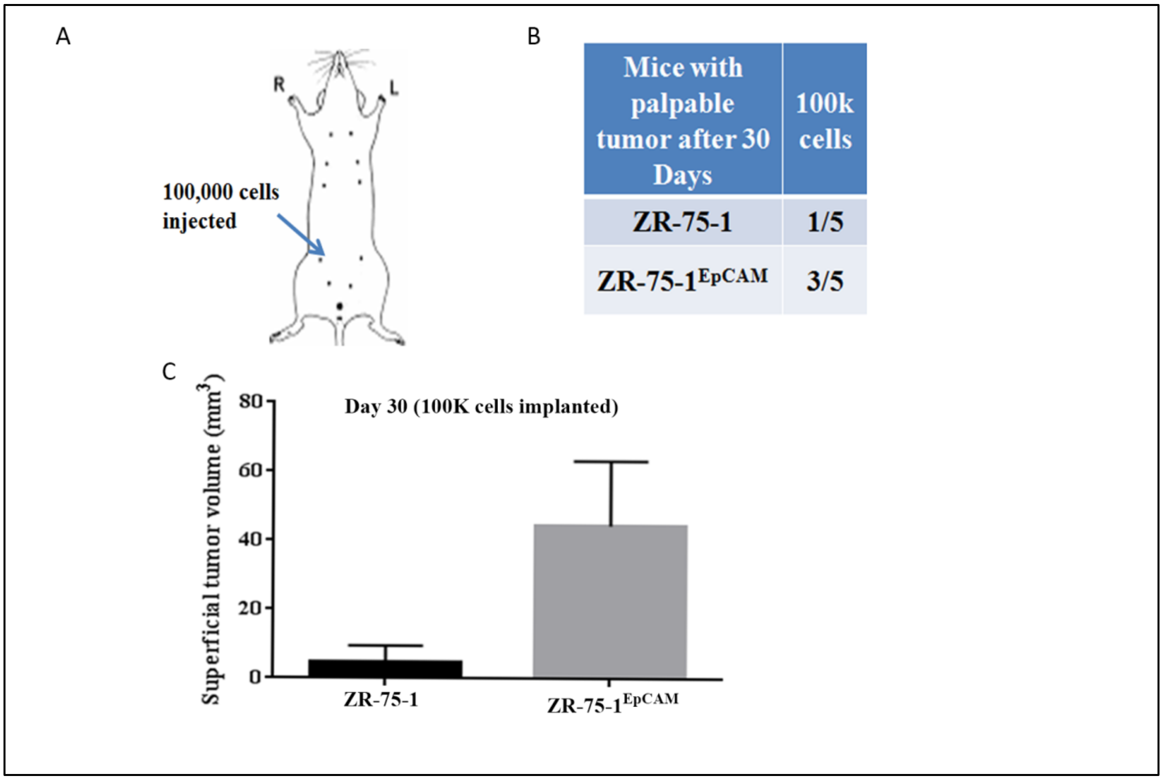

Supplementary Figure-6-A- *Diagram showing the injection of cells. 100,000 ZR-75-1 and ZR-75-1*^{EpCAM} *cells were injected in the mammary fatpad.* ***B***- *Table showing the number of mice in which there is palpable tumor after 30days.* ***C***- *Graph representing the volume of tumor (from panel B).*

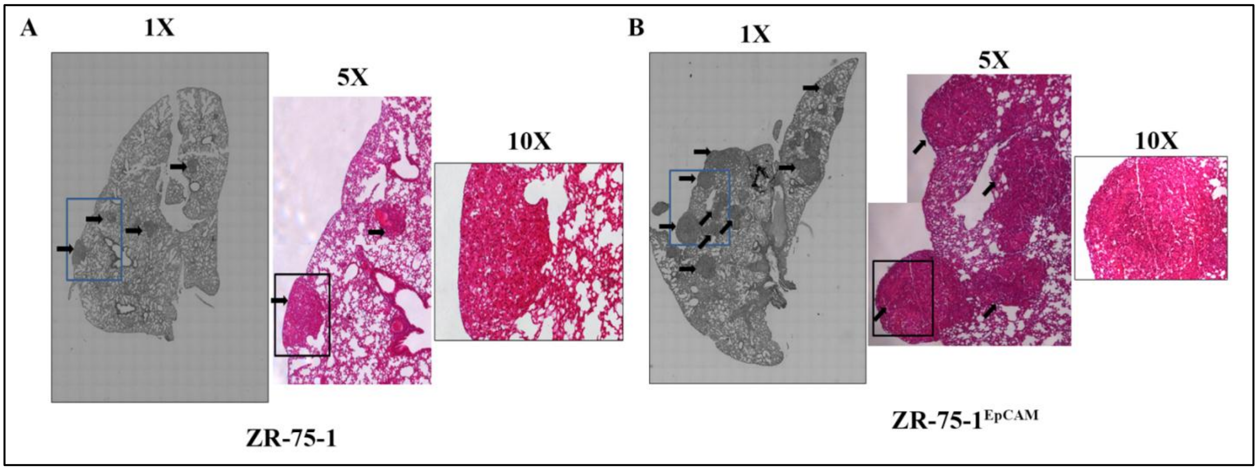

Supplementary Figure-7- *Representative photomicrograph image of lungs of mice belonging to ZR-75-1 tumor group* ***(A)*** *and ZR-75-1*^{EpCAM} *tumor group* ***(B)****. The images are in 1X magnification. A part of 1X, which is marked with blue box, is further imaged at 5X*

magnification. A part of 5X image marked with black box was also further imaged at 10X magnification. The black arrows denote the secondary metastasis within the lung.
